# Mesoscale sequence feature modulates AID activity in antibody diversification: Mesoscale DNA feature facilitates SHM

**DOI:** 10.3724/abbs.2023145

**Published:** 2023-08-03

**Authors:** Jiayang Li, Hai-Qiang Dai

**Affiliations:** State Key Laboratory of Molecular Biology Shanghai Institute of Biochemistry and Cell Biology Center for Excellence in Molecular Cell Science Chinese Academy of Sciences University of Chinese Academy of Sciences Shanghai 200031 China

The adaptive immune system produces diverse antibodies against a wide range of invading pathogens. Three mechanisms contribute to antibody diversification [1]. During early B cell development, V(D)J recombination assembles *immunoglobulin* ( *Ig*) genes by joining the variable (V), diversity (D) and joining (J) gene segments to form a large primary antibody repertoire. After antigenic challenge, activated B cells perform class switch recombination (CSR) to change the *Ig* heavy chain constant regions ( *IgH*) to expand the scope of immune response. Concurrent with CSR, somatic hypermutation (SHM) modifies *Ig* heavy and light chain variable regions ( *IgV*) to improve antigen-binding affinity and produce high-affinity antibodies [2].

Both CSR and SHM occur in activated germinal center B cells and are initiated by activation-induced cytidine deaminase (AID) [3]. The mutagenic activity of AID is mainly confined to switch (S) regions and *IgV* regions. SHM introduces further mutations in the *IgV* exons to allow affinity maturation, which is at the center of the humoral immune response to counter infections [2]. Why these mutations are largely concentrated in the non-consecutive complementarity-determining regions (CDRs) of *IgV* remains an enigma [4]. The groundbreaking study by Wang and colleagues [5], recently published in *Cell*, shed some light on this long-standing question that has puzzled antibody researchers for more than 40 years. It demonstrats that the flexible single-strand DNA (ssDNA) feature is the key to determine preferential hypermutability by increasing binding to the surface-charged patches of AID and facilitating deaminase activity.

## CDR Hypermutation Is an Evolutionary-conserved Feature Influenced by AID Activity and DNA Sequence *per se*

**Figure 1:**
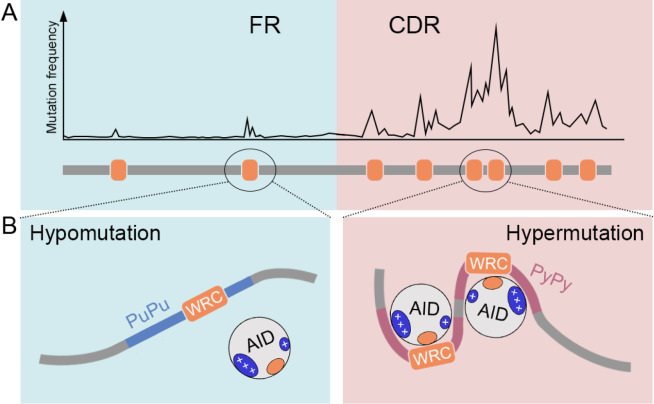
Figure 1 Mesoscale DNA feature facilitates somatic hypermutation (A) An illustrative artificial dataset shows the higher mutation frequency in WRC motifs in CDR-vs.-FR. (B) Model for flexible ssDNA feature in facilitating AID activity. Flexible pyrimidine-pyrimidine (Py-Py) bases frequently flank WRC motifs in CDR. Wang et al. [5] found that mesoscale DNA sequences exhibit strong binding affinity to the positively charged surface patches of AID, resulting in a preferential enhancement of deamination activity.

The CDRs are three short spaced hypervariable intervals located in the *IgV* exons, which are flanked by four relatively stable regions, termed as framework regions (FRs). The CDRs undergo a higher frequency of mutations than the FRs ( Figure 1A). This CDR-preferential hypermutation feature mainly relies on the predisposition of mutations, as evidenced by similar patterns observed in the intrinsic hypermutation profiles from non-productive V exons [4]. While the analysis of SHM hotspots has demonstrated the preferential targeting of cytidines (Cs) by AID in the context of WRCY or the complementary RGYW motif (W=A/T, R=A/G, Y=C/T), it is important to note that only a limited number of *IgV* sequences have been examined for their intrinsic SHM profiles [ 6, 7]. In a recent paper published in *Cell*, by utilizing a vast database of SHM profiles of the non-functional *Ig* heavy chain variable region ( *IgV*_H_) sequences, Wang *et al*. [5] demonstrated that the CDRs exhibit a great abundance of WRC motifs than the FRs. Crucially, they also established that cytidines in WRC motifs undergo mutations at a higher frequency in CDRs than in FRs.

To explore the molecular determinants behind CDR preference, Wang *et al* . [5] constructed an *in vitro* AID deamination assay containing purified AID and ssDNA substrates that performs mimicable SHM profiles comparing to *in vivo* hypermutation patterns. They found that the *in vivo* CDR-preferential hypermutation profiles could be recapitulated in an *in vitro* AID deamination assay on ssDNA. This remarkable discovery demonstrated that both AID and ssDNA sequence *per se* possess the ability to determine preferential hypermutability of WRC in CDR-vs.-FR. Furthermore, Wang *et al*. [5] extended the biochemical assay to analyze *in vitro* AID deamination profiles from 27 species, and they successfully established that the CDR-preferential hypermutability is highly evolutionary-conserved in tetrapod species. However, intriguingly, this general trend does not hold true for horses and GALT (gut-associated lymphoid tissue) species. In addition, Wang *et al*. [5] further demonstrated that the mutation frequencies of WRC in CDR3s are higher than that in the CDR1/2s, although the sequence features of CDR3 are either inherited from D/J segments or acquired through V(D)J recombination.

## DNA Sequence Affects SHM through Electrostatic Interactions between Mesoscale Adjacent Sequence of WRC Motifs and AID

An intricate network is applied to precisely regulate the efficacy of AID due to its DNA-damaging potential. While regulatory mechanisms are manipulated across multiple scales, ranging from nuclear large-scale to WRC motif microscale, the mesoscale level (5‒50 bp) has not received adequate attention [8]. Using the powerful *passenger-Ig* mouse model system [4], Wang *et al*. [5] demonstrated that WRC surrounding sequence contexts contribute to the WRC mutability *in vivo*, yet the WRC position *per se* does not determine mutability. They extended the *passenger-Ig* mouse model system by integrating CRISPR/Cas9-mediated CDR3 editing. Through the analysis of the SHM profiles in these models, they successfully established that the mesoscale sequence surrounding the WRC motifs contributes to the adjacent WRC mutability *in vivo* and plays a direct role in regulating AID deaminase activity at the ssDNA level.

AID interacts with ssDNA via a bifurcated substrate-binding surface, capturing two structured adjacent ssDNAs, one of which is identified as a substrate channel and the other as a assistant patch [9]. Wang *et al*. [5] hypothesized that AID could “sense” the mesoscale sequence feature adjacent WRC motifs via these surface patches. Using a combination of *in vitro* AID deamination assay, molecular dynamics simulations and single-molecule biochemistry, they demonstrated that AID surface patch-mediated interaction may drive the mesoscale preference. This interaction could potentially play a role in determining the preferential deamination based on electrostatic interactions between AID and ssDNA backbone. Moreover, the ssDNA base sequence might indirectly impact the binding process.

## DNA Strand Flexibility Contributes AID Deamination Preference in a Non-coding Way

DNA flexibility is a sequence-dependent conformational property. Previous reports showed that poly(dA) exhibits high rigidity, while poly(dT) displays remarkable flexibility [10]. The use of homopolymer-context substrates allows for the examination of how mesoscale DNA flexibility influences AID activity. In their study, Wang *et al*. [5] conducted experiments using a panel of substrates with increasing rigidity on either side of the AGCT motif, and demonstrated that the DNA region immediately 5′ to the AID target directly binds to AID via the charged patch. They also established that AID-favored substrates, which are enriched in pyrimidine-pyrimidine (Py-Py) motifs and characterized by weak stacking strength and high flexibility, demonstrate high mutability; whereas AID-disfavored substrates, which are enriched in purine-purine (Pu-Pu) motifs and have strong stacking strength and low flexibility, exhibit low mutability ( Figure 1B). During the evolution of antibody genes, the DNA sequence encoding the CDRs has acquired the feature of high flexibility, and insertion of a synthetic flexible motif adjacent to a cold-WRC motif renders it highly mutable [5]. Collectively, the study revealed that the AID sequence preference is modulated by the ssDNA flexibility, uncovering a non-coding role of the mesoscale DNA sequences in the CDRs in promoting SHM ( Figure 1B) and offering insights into the underlying mechanisms behind hypermutation patterns observed in B cell lymphoma.

In summary, Meng and colleagues [5] have successfully solved a long-standing question in the field of SHM. Their findings have profound implications for our understanding of the intricate mechanisms underlying *IgV* sequence evolution and AID *non-Ig* off-targeting preference. These discoveries provide valuable new insights into the future development of advanced humanized antibody animal models. Nevertheless, several intriguing questions still warrant further consideration. These include: 1) understanding the acquisition process of the mesoscale feature in CDR of *IgV* during evolution; 2) unraveling the unresolved mechanisms behind the acquisition of these features in somatic-assembled CDR3; 3) elucidating the influence of DNA secondary structure on flexibility *in vivo*; and 4) obtaining additional genetic evidence to comprehend the impact of the mesoscale feature on AID-initiated genome instability in cancer.
